# Neurological Insights into 16p11.2- And 22q11.2-Related Disorders: A Mini-Review

**DOI:** 10.2174/0113892029338299241211063307

**Published:** 2025-01-24

**Authors:** Yung-Hsiu Lu, Yann-Jang Chen, Shan-Ju Lin, Ting-Rong Hsu, Dau-Ming Niu, Wei-Sheng Lin

**Affiliations:** 1 Department of Pediatrics, Taipei Veterans General Hospital, Taipei, Taiwan;; 2 Institute of Clinical Medicine, School of Medicine, National Yang Ming Chiao Tung University, Taipei, Taiwan;; 3 Department of Life Sciences and Institute of Genome Sciences, National Yang Ming Chiao Tung University, Taipei, Taiwan;; 4 Department of Physical Medicine and Rehabilitation, National Taiwan University Hospital Yunlin Branch, Yunlin, Taiwan;; 5 School of Medicine, National Yang Ming Chiao Tung University, Taipei, Taiwan

**Keywords:** Autism, copy number variant, epilepsy, intellectual disability, neurodevelopmental disorder, neuropsychiatric disorder, schizophrenia

## Abstract

Copy Number Variations (CNVs) involving 16p11.2 or 22q11.2 are often linked to neurodevelopmental and neuropsychiatric disorders, including autism spectrum disorder, attention deficit hyperactivity disorder, cognitive impairment, epilepsy, and schizophrenia. The pathogenetic mechanisms underlying these neurological phenotypes remain incompletely understood, partly due to the multitude of genes involved and the complex gene-gene interactions at these loci. Nonetheless, recent advances in experimental technology and bioinformatics have greatly enhanced our understanding of the neurobiology of 16p11.2- and 22q11.2-related disorders. Herein, we aim to provide an updated mini-review on neurological aspects of these disease-associated CNVs, with emphasis on clinical and mechanistic insights as well as potential therapeutic implications.

## 1 INTRODUCTION

Copy Number Variations (CNVs) are a common form of structural variations in the human genome. Some CNVs appear to be benign (*i.e*., without apparent clinical consequences), while others are implicated in disease susceptibility. Among disease-associated CNVs, 16p11.2 and 22q11.2 represent two loci that are often linked to neurodevelopmental and neuropsychiatric disorders, including Autism Spectrum Disorder (ASD), Attention Deficit Hyperactivity Disorder (ADHD), cognitive impairment, epilepsy, Schizophrenia (SCZ), bipolar disorder, and a variety of neuroanatomical phenotypes [1, 2]. The neurological symptoms could have significant impacts on the functioning and quality of life in patients carrying these CNVs [3].

Because multiple genes (~30 genes in the 16p11.2 locus and 40-50 genes in the 22q11.2 locus) are involved simultaneously, it has been challenging to decipher the neurobiological mechanisms underlying the diverse neurological and psychiatric manifestations in 16p11.2- and 22q11.2-related disorders. Nonetheless, advances in experimental technology and bioinformatics have enabled more in-depth research to dissect the pathophysiological underpinnings of neurological disturbances in these conditions. Herein, we aim to summarize recent neurological insights into these CNVs in this mini-review, which is not intended to be comprehensive but is focused on selected topics of clinical or neurobiological relevance.

## 2 NEUROLOGICAL CONDITIONS ASSOCIATED WITH 16p11.2 AND 22q11.2 CNVS

An overarching feature regarding the spectrum of neurological manifestations associated with 16p11.2 and 22q11.2 CNVs is phenotypic variability, which could be attributed to pleiotropy, variable expressivity, and incomplete penetrance [2, 4]. Some carriers may be apparently asymptomatic, while others may be affected by different combinations of various neurodevelopmental or neuropsychiatric conditions, including ASD, ADHD, intellectual disability, epileptic seizures, SCZ, and bipolar disorder (Table **1**). These neurological manifestations, albeit relatively nonspecific, could serve as clues to the genetic diagnoses, particularly in the presence of relevant abnormalities in other organ systems [5].

### 2.1 Autism Spectrum Disorder (ASD)

Both 16p11.2 and 22q11.2 are listed as susceptibility loci for ASD. For 22q11.2 CNVs, it is interesting to note that both deletion and duplication are associated with an increased risk of ASD [4, 6] despite opposite directions of gene dosage changes. Likewise, both deletion and duplication of 16p11.2 predispose individuals to ASD [7-10], despite opposite directions of gene dosage and head size changes (see sections 2-6). In a clinically ascertained cohort involving 85 persons with 16p11.2 deletion, 24% had a diagnosis of ASD, and those not meeting the criteria for ASD had a higher prevalence of autistic traits such as repetitive and stereotyped behaviors and social difficulties [11]. These socio-cognitive deficits might be mediated by reduced functional connectivity with the prefrontal cortex, as demonstrated in human carriers and mouse models of 16p11.2 deletion [12].

Several genes within these CNV loci have been linked to ASD or related phenotypes. For example, Richter *et al.* reported that mice with loss of *Taok2* (see section 3-2) displayed reduced social behavior [13]. Catechol-O-methyltransferase (COMT) and proline dehydrogenase/oxidase 1 (PRODH) (see section 3-5), both encoded by genes at the 22q11.2 locus, have also been implicated in ASD phenotype [14].

### 2.2 Attention Deficit Hyperactivity Disorder (ADHD)

Both 16p11.2 duplication and deletion are associated with a higher risk of ADHD [7], with an odds ratio of 7.0 and 4.0, respectively, in one study involving European and US children [10]. Both 22q11.2 duplication and deletion are also associated with an increased risk of ADHD or related traits [2, 6, 15]. Microduplications involving 22q11.2 and 16p11.2 were found to be significantly overrepresented among patients diagnosed with comorbid ASD and ADHD [16]. Neuroimaging studies could potentially unveil the neural substrates underlying attention deficits. For example, a structural MRI study in children with 22q11.2 deletions showed localized thinning in the ventromedial occipital-temporal cortex and the anterior cingulate, two areas related to attentional control (see section 2-6) [17].

### 2.3 Intellectual Disability

A recent review reported a prevalence of intellectual disability in 10.3-28.1% of 16p11.2 deletion carriers and 30.5-40.3% of duplication carriers, although non-clinical 16p11.2 CNV carriers might be underrepresented in those surveys [7]. De novo 16p11.2 deletion carriers had, on average, a 27-point decrement of full-scale IQ as compared to their parents, whereas there was enormous heterogeneity in this phenotype [11, 18]. Using large genotyped cohorts, Vysotskiy *et al.* found that the *SPN* gene at 16p11.2 was strongly associated with IQ [19].

Both 22q11.2 duplication and deletion are also associated with an increased risk of intellectual disability or related trait [4], and most patients with 22q11.2 deletion syndrome (22q11.2DS, also known as velocardiofacial or DiGeorge syndrome) have borderline to mild intellectual disability [20]. De Koning *et al.* reported an association of *PRODH* genotype with full-scale IQ in adults with 22q11.2DS [21].

### 2.4 Epileptic Seizures

Reciprocal CNVs at 16p11.2 and 22q11.21 have been identified as genome-wide significant susceptibility loci for seizure disorders [2]. Epileptic seizures are common in people with 16p11.2 CNVs, and the majority of studies to date have shown that one-sixth or more people with 16p11.2 deletions or duplications have been diagnosed with epilepsy or seizure disorders [7, 22]. In addition, 16p11.2 has been identified as a risk locus for juvenile myoclonic epilepsy, with *STX1B* and *CACNA1I* being implicated as the genes potentially responsible for the association [23, 24]. It is notable that *Mvp*+/−; *Mapk*3+/− mice (but not *Mvp*+/− or *Mapk*3+/− mice) exhibited significantly reduced seizure duration in a pentylenetetrazol-induced seizure paradigm [25], suggesting that dosage changes across multiple genes in this locus have very complicated effects on seizure susceptibility.

Epileptic seizures are also common in patients with 22q11.2DS [26]. In a study involving 108 young people (mean age 13.6 years) carrying 22q11.2 deletion, 11% (12/108) had a diagnosis of epilepsy. In addition, 59.4% (57/96) of those without a diagnosis of epilepsy had seizures or seizure-like symptoms, which was significantly higher than that in sibling controls (13.3%, 8/60). People with 22q11.2 deletion are also prone to febrile seizures (24.1%, 26/107). In another study involving 202 adult patients (mean age 39.3 years) carrying 22q11.2 deletion, 15.8% (32/202) had a history of seizures, and 4% (9/202) met the diagnostic criteria for epilepsy [27]. The remaining 23 patients with a history of seizures had acute symptomatic seizures, which were usually associated with hypocalcemia and/or antipsychotic or antidepressant exposure. The seizure types were diverse, with generalized tonic-clonic seizures being the most common. A recent phenome-wide association study (PheWAS) suggested that 22q11.21 CNVs had differential epilepsy-type associations, with deletion and duplication carriers tending to have generalized and focal epilepsies, respectively [2]. By contrast, an earlier study reported that focal epilepsy was more common than generalized epilepsy, while either occurred in a significant proportion (more than one-fourth) of patients with 22q11.2DS [28]. Taken together, 22q11.2 deletion seems to be associated with an increased susceptibility to epileptic seizures across the majority of lifespan.

### 2.5 Psychosis

Both 22q11.2 and 16p11.2 are implicated in schizophrenia (SCZ) in genome-wide studies [1]. Specifically, the associations of 22q11.21 deletions and proximal (BP4–BP5) 16p11.2 duplications with SCZ have been well established [29], and distal 16p11.2 deletions are also associated with an increased risk of SCZ. Generally, the odds ratio for these SCZ-associated CNVs is larger as compared to that for the SCZ-associated single-nucleotide variants with similar minor allele frequencies [30]. 22q11.2 deletion is also implicated in more broadly defined psychosis, with a peak age at onset in late adolescence [31]. It has been suggested that children and adolescents with a diagnosis of psychotic disorders be screened for CNVs [32]. Conversely, several studies have shown that 22q11.2 duplication confers protection against SCZ [33, 34], although this was not found in a recent report [9].

Vysotskiy *et al.* found several significant gene-trait pairs, including *NPIPB11* and *SLX1B* (both at 16p11.2) and psychosis, in a PheWAS [19]. They also found an association between *SCARF2* (at 22q11.21) and mood disorders. Using large genotyped cohorts, they found individual genes at 16p11.2 associated with SCZ (*TMEM219*, *INO80E*, *YPEL3*); upregulation of *INO80E* was then identified as the driver of SCZ using conditional analysis [19]. In neuronal models differentiated from induced pluripotent stem cells (iPSC) derived from 22q11.2DS patients, the application of antipsychotic drugs rescued the functional defects in calcium signaling and electrophysiology [35].

### 2.6 Neuroanatomical Phenotypes

Although 22q11.2DS is not associated with major defects in corticogenesis in patient-derived models [35], abnormal neuroimaging findings are common in patients with 22q11.2DS [26]. Some abnormalities are acquired in nature, yet congenital malformations of the brain have also been documented and may be the pathological basis of other neurological manifestations [27]. The loss of *Dgcr2*, *Dgcr8*, and *Ranbp1* has been associated with impaired corticogenesis in mice [36-38], and gray matter heterotopia and polymicrogyria seem to be more common in 22q11.2DS [20, 28, 39]. Dedicated structural imaging studies have revealed localized thinning in the ventromedial occipital-temporal cortex and anterior cingulate, which are implicated in visuospatial processing and attentional control in children/adolescents with 22q11.2DS [17]. A recent study showed that 22q11.2 copy number is positively correlated with hippocampal volume, suggestive of gene dosage effects [40].

Brain morphometric studies have largely shown a moderate to large effect size of 16p11.2 or 22q11.2 CNVs on global and regional measures [41, 42]. However, there is usually no significant correlation between morphometric and clinical measures, exemplifying the complex interrelationships between genes, brain structures, and behaviors. Kundu *et al.* reported that neuroimaging patterns specific for 16p11.2 CNVs could be revealed by a novel analytical technique based on generative machine learning [43]. Their findings suggested that the penetrance of 16p11.2 CNVs is high in terms of the effects on brain structures, and the neuroimaging patterns identified may serve as endophenotypes for future research and clinical applications.

It has been firmly established that 16p11.2 copy number is inversely correlated with brain size, and its effects on brain structures appear to be pervasive in human studies [44]. Both mouse models and patient-derived cortical organoids recapitulated these brain-size phenotypes [45, 46]. *KCTD13* was considered the major molecular driver of this phenotype, as its overexpression and suppression yielded microcephaly and macrocephaly, respectively, in zebrafish models [47]. However, subsequent studies in mice did not replicate the findings [48-50]. Another study reported that *Taok2* gene dosage is inversely correlated with brain volume in mice [13], suggesting that *TAOK2* might be the driver of head size phenotype. Furthermore, *Taok2* knockout (but not *Kctd13* knockout) mice exhibited structural alterations in the brain similar to that observed in heterozygous 16p11.2 microdeletion mice [50]. Notably, the effects of *Taok2* deficiency on the brain structures are region-specific in mouse studies [13, 50].

In addition to altered brain size, *Taok2* deficiency in mice was associated with developmental defects in the cerebral cortex. TAOK2, specifically the isoform TAOK2α, ameliorated the neuronal migration defects in the heterozygous 16p11.2 microdeletion mouse model [50]. Taken together, these findings indicated that *TAOK2* is likely an important player in neuroanatomical phenotypes in 16p11.2 CNVs. On the other hand, multiple different genes at 16p11.2 also likely contribute to various neuroanatomical phenotypes, often in a sex-specific manner, as demonstrated by systematic studies in mouse models [25]. Further studies may be needed to clarify whether sex differences in neuroanatomical features are present in human carriers of 16p11.2 CNVs.

## 3 ROLES OF INDIVIDUAL GENES AND ASSOCIATED PATHWAYS IN 16p11.2 AND 22q11.2 CNVS

16p11.2 and 22q11.2 CNVs involve dozens of genes. Individual genes within these CNV regions, particularly the dosage-sensitive ones, might play dominant roles in the expression of some phenotypes. For example, *TBX1* haploinsufficiency may be the main driver for the cardiovascular phenotype in 22q11.2DS, and evidence from animal and human studies suggests that it also contributes to some neurological and/or psychiatric phenotypes [51-53]. Many genes at 16p11.2 and 22q11.2 are implicated in neurodevelopmental processes [54, 55], and their roles are being unveiled. Particularly noteworthy is the importance of gene-gene interactions [25, 56, 57], which might come in several flavors [54, 58]. On the other hand, different genes often converge on common signaling pathways. The following is a concise review of recent progress in the molecular neurobiology of 16p11.2 and 22q11.2 CNVs.

### 3.1 KCTD13, and KCTD13-Cul3-RhoA and KCTD13-ERBB Pathways

The role of KCTD13 in head size, as discussed in sections 2-6, may involve complex genetic interactions and remains to be elucidated. Investigations of the network dynamics of 16p11.2 protein interactions implicated the KCTD13-Cul3-RhoA (Ras homolog gene family member A) pathway in neurological phenotypes [59]. Increased levels of RhoA, a KCTD13/CUL3 ubiquitin ligase substrate, have been shown to be correlated with reduced synaptic transmission in *Kctd13* deletion mice [49]. Object recognition memory deficit, a robust phenotype associated with 16p11.2 deletion mice, was restored by administration of fasudil (an inhibitor of RhoA signaling) in adult *Kctd13* deficient and 16p11.2 deletion mouse models [60]. On the other hand, KCTD13 deficient cortical neurons derived from human iPSCs showed decreased neurite formation, which was neither associated with RhoA accumulation nor rescued by inhibitors of RhoA signaling [61]. Instead, the impaired neurite formation was rescued by activating ERBB kinases, suggesting that altered ERBB signaling might contribute to some neurological phenotypes in carriers of 16p11.2 CNVs.

### 3.2 TAOK2, and TAOK2-JNK1 and TAOK2-RhoA Pathways

TAOK2 is a serine/threonine kinase, and it directly phosphorylates the cytoskeletal GTPase Septin7, which in turn mediates PSD95 stability and dendritic spine maturation [62]. Recent studies showed that *Taok2* knockout and heterozygous 16p11.2 microdeletion mouse models shared similar neuronal migration defects [50]. The heterozygous 16p11.2 microdeletion mouse model displayed reduced levels of phosphorylated JNK1, and activation of the Taok2-JNK1 signaling axis rescued the neuronal migration defects [50]. Loss of Taok2 activity also led to reduced RhoA activation and pharmacological activation of RhoA rescued synaptic phenotypes in the *Taok2* knockout neuronal model [13]. Together with other studies discussed in section 3-1, RhoA appears to be a hub in the signaling network downstream of different molecules encoded within the 16p11.2 locus. There seems to be an optimal level of RhoA activity for neuronal connectivity, as both too much and too little of it have been linked to synaptic deficits [13, 49]. It is interesting to note that Kctd13 and Taok2 deficiency are associated with RhoA changes in opposite directions [13, 49], and that both 16p11.2 deletion and duplication are associated with elevated levels of the active form of RhoA in patient-derived cortical organoids [45].

### 3.3 Major Vault Protein, MAPK3, and ERK Signaling

Major vault protein (MVP), encoded by *MVP* at 16p11.2, is the major protein component of the vault, a mysterious cellular structure comprising proteins and RNAs. *Mvp* has been shown to interact with *Kctd13* in a sex-specific manner in mice [48]. Murine studies showed that the small noncoding vault RNA (vtRNA), another component of the vault, is released from the vault in neurites with the help of phosphorylated MVP [63]. The vtRNA then binds to and activates MEK1, thereby enhancing extracellular signal-regulated kinase (ERK) activation and synapse formation. ERK1, also known as mitogen-activated protein kinase 3, is encoded by *MAPK3*, another gene at 16p11.2. *MAPK3*/ ERK1 has been identified as the most topologically important hub in protein-protein interaction networks within the 16p11.2 region and broader gene networks of SCZ-associated CNVs [64]. Pharmacological inhibition of ERK signaling reversed the dendritic alternations observed in a neuronal model of 16p11.2 duplication [64]. Other genes at 16p11.2, such as *TAOK2* and *SEZ6L2*, might also affect ERK signaling *via* Septin7 [62, 65] and protein kinase C [66], respectively. Despite multiple lines of evidence implicating the role of ERK signaling in 16p11.2 CNVs, preliminary studies indicated that ERK activity is not associated with neuroanatomical phenotypes in this context [25].

### 3.4 PRRT2

The roles of *PRRT2* (at 16p11.2) as a disease-causing or disease-predisposing gene have been established for a variety of neurological conditions, and both reduced and increased gene dosage are associated with neurological phenotypes [67, 68]. The dosage-sensitive nature of PRRT2 is further illustrated by a recent mouse study, which showed that increased glutamatergic tone and seizure susceptibility in 16p11.2 duplication mice were ameliorated by *Prrt2* dosage correction [68]. Other genes at 16p11.2 might modulate the effects of PRRT2 deficiency, as the frequency of paroxysmal kinesigenic dyskinesia in 16p11.2DS (<9% [69]) appears to be much lower than its penetrance in *PRRT2* mutation carriers (>60% [67]). PRRT2 is likely a polyfunctional and multitasking molecule critical for neural circuit operations. More detailed information is beyond the scope of this review and can be found elsewhere [67].

### 3.5 COMT, PRODH, and Hyperprolinemia

*PRODH* (at 22q11.2), an evolutionarily conserved gene, codes for a mitochondrial enzyme known as proline dehydrogenase or proline oxidase, which plays a catalytic role in the conversion of proline to pyrroline-5-carboxylate [55, 70]. Pyrroline-5-carboxylate can be further metabolized to glutamate, and glutamatergic neurotransmission is altered in Prodh-deficient mice [71]. PRODH deficiency causes hyperprolinemia type 1 in humans, which is associated with autistic features, hyperactivity, cognitive impairment, seizure, and increased susceptibility to SCZ. Hyperprolinemia *per se* has been implicated in depression, which may be partly mediated through altered GABAergic neurotransmission [72, 73]. Proline may also interact with COMT (encoded by *COMT* at 22q11.2) genotype in modifying negative symptoms in both SCZ and bipolar disorder [74] and in modifying startle reactivity in 22q11.2DS [21]. Indeed, epistasis has been well recognized between *Prodh* and *Comt* at both transcriptional and behavioral levels in mouse models [71], and further research is worth pursuing to elucidate the clinical relevance of these two genes and their interactions.

### 3.6 Glutamatergic Neurotransmission and Other Neurotransmitter Systems

Both 16p11.2 and 22q11.2 contain genes that interact with metabotropic glutamate receptors (mGluRs), and duplications involving these mGluR interacting genes are found to be significantly associated with ADHD plus ASD [16]. PRODH deficiency is associated with altered glutamatergic neurotransmission, as mentioned in section 3-5. *DGCR2*, another gene at 22q11.2, encodes a cell adhesion molecule that is enriched in postsynaptic densities and is required for dendritic spine development [75]. Decreased hippocampal glutamatergic transmission was noted in DGCR2-deficient mice [75]. Conversely, increased glutamatergic tone has been demonstrated in 16p11.2 duplication mice. This may contribute to a predisposition to seizures, which is probably mediated by increased *Prrt2* gene dosage [68].

Other neurotransmitter systems, such as GABAergic [76] and dopaminergic circuits [71, 77], may also be implicated in some neurological phenotypes related to 22q11.2 and 16p11.2 CNVs.

## CONCLUSION & FUTURE PERSPECTIVES

It is obvious that phenotypic heterogeneity in subjects carrying 16p11.2 or 22q11.2 CNVs cannot be fully explained by individual gene dosage, and accumulating evidence indicates that epistasis may be another source that modulates the penetrance and expressivity [54, 71]. Advances in genetic manipulation have enabled more nuanced mechanistic dissection of disease-associated CNVs. For example, hemi-deletion of three genes (*Taok2*, *Sez6l2*, and *Mvp*) recapitulated some behavioral phenotypes of 16p11.2 hemi-deletion in mouse models. In contrast, an additional hemi-deletion of *Mapk3* paradoxically reduced the phenotypic similarity to 16p11.2 hemi-deletion [56]. On the other hand, male mice with hemi-deletion of both *Mvp* and *Mapk3* (but not *Mvp* knockout or *Mapk3* hemi-deletion alone) exhibited decreased anxiety-like traits [25]. These findings exemplify the complex genetic interactions at the 16p11.2 locus, which have been demonstrated to be pervasive in *Drosophila* models [54]. A network biology-based approach has proven to be useful in this regard [59, 64, 68]. More studies are needed to decipher the contributions of individual genes and their interactions with diverse phenotypes.

Research findings to date are not always consistent. For instance, 16p11.2 duplications were associated with greater deficits than 16p11.2 deletions in terms of complex cognition (including nonverbal reasoning and spatial processing) in human subjects [78]. By contrast, 16p11.2 deletion appeared to cause more severe neurological phenotypes than 16p11.2 duplication in mouse models [46]. Possible explanations include survival bias and ascertainment bias [11, 46]. Some noncoding genetic elements like microRNAs seem less conserved across species. Thus, they might account for some discrepancies between animal models and human carriers [70]. Other nongenetic modifiers, such as environmental factors, may also play a role in phenotypic expression and deserve further studies.

More sophisticated research approaches, such as complex *in vitro* models, single-cell omics, and spatial biology, may help further elucidate the neurobiological mechanisms of 16p11.2 and 22q11.2 CNVs. For example, genome-engineered or patient-derived iPSCs, which could be further differentiated into cellular models (such as dopaminergic neurons) or brain organoids, have been increasingly used to investigate these disorders in recent years [35, 45, 61, 77, 79-81]. These experimental advances not only yield mechanistic insights but also serve as testbeds for potential therapeutics. Inhibition of RhoA signaling has been shown to rescue the electrophysiological phenotype in the 16p11.2-deleted dopaminergic neuronal network model derived from human iPSC [77] and the neuronal migration defects in patient-derived brain organoid models [45]. Hopefully, these research endeavors could further our understanding of the polygenic influences on neurodevelopment and psychiatric disorders and ultimately lead to better care for patients with 16p11.2- and 22q11.2-related disorders.

## AUTHORS’ CONTRIBUTIONS

The authors confirm their contribution to the paper as follows: study conception and design: WSL; Writing the Paper: YHL, WSL; Data Analysis or Interpretation: YJC, WSL; Data Collection: SJL, WSL; Writing - Reviewing and Editing: TRH, DMN, WSL. All authors reviewed the results and approved the final version of the manuscript.

## Figures and Tables

**Table 1:** Neurodevelopmental and neuropsychiatric disorders associated with CNVs involving 16p11.2 or 22q11.2.

** - **	**16p11.2 del**	**16p11.2 dup**	**22q11.2 del**	**22q11.2 dup**
MIM number	# 611913	# 614671	# 188400 (DGS) # 192430 (VCFS)	# 608363
Intellectual disability	↑	↑	↑	↑
ASD	↑	↑	↑	↑
ADHD	↑	↑	↑	↑
Schizophrenia	~	↑	↑	↓ or ~
Epilepsy	↑	↑	↑	~?
Brain size	↑	↓	↓	↓?

**Note:** For quantitative details, such as the odds ratio for individual conditions, see references like [4, 7]. Limited data are available regarding some aspects of 22q11.2 dup. ↑, increased; ↓, decreased; ADHD, attention deficit hyperactivity disorder; ASD, autism spectrum disorder; del, deletion; DGS, DiGeorge syndrome; dup, duplication; VCFS, velocardiofacial syndrome.

## LIST OF ABBREVIATIONS

16p11.2DS: 16p11.2 Deletion Syndrome
22q11.2DS: 22q11.2 Deletion Syndrome
ADHD: Attention Deficit Hyperactivity Disorder
ASD: Autism Spectrum Disorder
BP: Breakpoint
CNV: Copy Number Variant (or Copy Number Variation)
COMT: Catechol-O-methyltransferase
ERK1: Extracellular Signal-regulated Kinase 1
iPSC: Induced Pluripotent Stem Cell
MAPK: Mitogen-activated Protein Kinase
mGluR: Metabotropic Glutamate Receptor
MVP: Major Vault Protein
PheWAS: Phenome-wide Association Analysis
PRODH: Proline Dehydrogenase
RhoA: Ras Homolog Gene Family Member A
SCZ: Schizophrenia
vtRNA: Vault RNA
